# Induced pluripotent stem cells from GMP-grade hematopoietic progenitor cells and mononuclear myeloid cells

**DOI:** 10.1186/scrt87

**Published:** 2011-11-16

**Authors:** Seiga Ohmine, Allan B Dietz, Michael C Deeds, Katherine A Hartjes, David R Miller, Tayaramma Thatava, Toshie Sakuma, Yogish C Kudva, Yasuhiro Ikeda

**Affiliations:** 1 Department of Molecular Medicine, College of Medicine, Mayo Clinic, 200 First Street S.W., Rochester, MN 55905, USA; 2 Human Cell Therapy, Department of Laboratory Medicine/Pathology, College of Medicine, Mayo Clinic, 200 First Street S.W., Rochester, MN 55905, USA; 3 Division of Endocrinology, College of Medicine, Mayo Clinic, 200 First Street S.W., Rochester, MN 55905, USA

## Abstract

**Introduction:**

The induced pluripotent stem cell (iPSC) technology allows generation of patient-specific pluripotent stem cells, thereby providing a novel cell-therapy platform for severe degenerative diseases. One of the key issues for clinical-grade iPSC derivation is the accessibility of donor cells used for reprogramming.

**Methods:**

We examined the feasibility of reprogramming mobilized GMP-grade hematopoietic progenitor cells (HPCs) and peripheral blood mononuclear cells (PBMCs) and tested the pluripotency of derived iPS clones.

**Results:**

Ectopic expression of OCT4, SOX2, KLF4, and c-MYC in HPCs and PBMCs resulted in rapid iPSC derivation. Long-term time-lapse imaging revealed efficient iPSC growth under serum- and feeder-free conditions with frequent mitotic events. HPC- and PBMC-derived iPS cells expressed pluripotency-associated markers, including SSEA-4, TRA-1-60, and NANOG. The global gene-expression profiles demonstrated the induction of endogenous pluripotent genes, such as *LIN28*, *TERT*, *DPPA4*, and *PODXL*, in derived iPSCs. iPSC clones from blood and other cell sources showed similar ultrastructural morphologies and genome-wide gene-expression profiles. On spontaneous and guided differentiation, HPC- and PBMC-derived iPSCs were differentiated into cells of three germ layers, including insulin-producing cells through endodermal lineage, verifying the pluripotency of the blood-derived iPSC clones.

**Conclusions:**

Because the use of blood cells allows minimally invasive tissue procurement under GMP conditions and rapid cellular reprogramming, mobilized HPCs and unmobilized PBMCs would be ideal somatic cell sources for clinical-grade iPSC derivation, especially from diabetes patients complicated by slow-healing wounds.

## Introduction

Because embryonic stem (ES) cells can self-renew indefinitely and differentiate into any cell present in the adult organism, ES cells provide a unique platform for regenerative medicine approaches. In early 2009, the US Food and Drug Administration (FDA) approved the first clinical trial using ES cells in patients with spinal cord injuries. Although the FDA temporarily placed the trial on hold because of concerns over the risk of ES-derived cyst formation, the clinical hold was lifted, and the first patient for ES cell treatment was enrolled by Geron in late 2010 [1]. The use of ES-derived, terminally differentiated retinal pigment epithelium cells for Stargardt macular dystrophy and dry age-related macular degeneration was also approved by the FDA, and Advanced Cell Technology is currently enrolling patients [2]. Despite these advances in clinical applications, the use of ES cells is associated with ethical issues and immunologic mismatch, which could affect their widespread use in the clinic.

The induced pluripotent stem cell (iPSC) technology allows generation of autologous pluripotent stem cells without using an embryonic cell source. Expression of the transcription factors OCT3/4, SOX2, KLF4, and c-MYC [3-7] or OCT-3/4, SOX2, NANOG, and LIN28 [8] in somatic cells results in iPSCs, which have phenotypes very similar to those of ES cells, including the morphology, self-renewal, and pluripotent potentials, and expression of pluripotency-associated factors, including SSEA-4 and TRA-1-60. Global gene-expression analysis of human iPSCs has also revealed patterns similar to those of human ES cells, with notable upregulation of pluripotency-associated genes such as *OCT4*, *SOX2*, *NANOG*, *TERT*, and *DPPA4 *[3-5,8]. Similar to ES cells, iPSCs can be differentiated into various cell types, such as insulin-producing cells [9-11], neurons [12], cardiomyocytes [13-15], and cells of hematopoietic lineages [13]. Various types of somatic cells have been successfully reprogrammed, including fibroblasts, stomach and liver cell cultures [16], human keratinocytes (HKs) [17,18], frozen human monocytes [19], cord blood cells [20-22], and blood cells [23,24], including mature B [25-28] and T lymphocytes [29-32]. For clinical applications, it would be ideal if iPSCs could be generated from somatic cells through a minimally invasive tissue-procurement procedure under GMP-compliant process. In this regard, blood cells are one of the most promising cell sources for clinical-grade iPSC derivation.

Here we examined the feasibility of iPSC derivation from GMP-grade mobilized hematopoietic progenitor cells (HPCs) and unmobilized peripheral blood mononuclear cells (PBMCs), and characterized derived iPSCs for global gene-expression profiles and pluripotency.

## Materials and methods

All studies were approved by the Institutional Review Board and Institutional Animal Care and Use Committee.

### Cells

GMP-grade HPCs were originally harvested from patients for stem cell therapy. Specifically, HPCs were harvested after mobilization by injection with granulocyte colony-stimulating factor for 5 days in the Human Cellular Therapy Laboratory at the Mayo Clinic. Those cell products are routinely discarded as clinical wastes when patients have died before the initiation of stem cell therapy. We received de-identified clinical-waste HPCs from the Human Cellular Therapy Laboratory for iPSC derivation. The use of clinical-waste HPCs for iPSC generation was approved by the Institutional Review Board, including the Biospecimens Subcommittee. For mononuclear myeloid cell derivation, 10 ml of whole blood was purchased and used to isolate PBMCs, as reported previously [33,34].

### Lentiviral vector production

Pluripotency-associated factor-expressing lentiviral vectors, pSIN-OCT4, pSIN-SOX2, pSIN-KLF4, and pSIN-cMYC were described previously [12]. These vectors were produced by transient transfection of 293T cells. Vector titers were determined with immunostaining [12].

### iPSC derivation

HPCs and PBMCs were cultured overnight in StemSpan H3000 serum-free medium (StemCell Technologies, Vancouver, Canada), which contains only human-derived or recombinant human proteins, supplemented with StemSpan CC100 cytokine cocktail (StemCell Technologies). Cells (10^5 ^cells/500 μl of medium in each well) in uncoated wells of a 24-well plate were then transduced with four stemness factor-expressing lentiviral vectors overnight. One third of the culture supernatants were carefully removed and replaced daily with H3000 growth medium supplemented with CC100 cytokine cocktail. Three days after vector infection, cells in a well of a 24-well plate were transferred to a Matrigel (BD Biosciences, Bedford, MA)-coated well of a 6-well plate. Starting 5 days after vector infection, cells were maintained in HEScGRO medium (100 ml; Millipore, Billerica, MA) supplemented with mTeSR-1 maintenance media (25 ml; StemCell Technologies) [11]. 7 to 10 days after vector infection, the reprogrammed cells began to form colonies with iPS morphology. At 2 to 3 weeks after vector infection, cultures were treated with Cell Dissociation Buffer (Invitrogen, Grand Island, NY) for 5 to 10 minutes to help lift clones, and individual iPSC-like clones were carefully picked up with a P200 pipette, and placed into Matrigel-coated wells in a 96-well plate. To prevent spontaneous differentiation, the iPSC culture medium was replaced daily, and differentiated cells in the cultures were manually removed with a pipette tip. As the clones grew, cultures were expanded into larger culture plates for further characterization. Clones were preserved by using Xeno-FREEze Human Embryonic Stem Cell Freezing Medium (Millipore). A verified iPSC clone, HCF1, from primary human fibroblast (HCF) cells, was described previously [11]. Primary human keratinocytes and keratinocyte-derived iPSC clones were also used as controls [35].

### Immunostaining and alkaline phosphatase staining

For immunostaining of iPSC, cells were fixed for 20 minutes at room temperature in 4% paraformaldehyde solution in PBS, washed several times in PBS, and blocked for 30 minutes in PBS with 5% fetal bovine serum. Cells were then stained with primary antibodies overnight at 4°C, rinsed with PBS, and incubated with secondary antibodies for 1 hour at RT. For immunostaining of differentiated cells, cells at different stages of differentiation were fixed and stained with primary and secondary antibodies. Primary antibodies used for characterization of iPSC and iPSC-derived cells were SSEA-4 and TRA-1-60 (Millipore, #SCR001), OCT4 (Cell Signaling Technology, #2750, Danvers, MA), NANOG (Abcam, #ab21624, Cambridge, MA), mouse anti-SOX17 (R&D Systems, #MAB1924, Minneapolis, MN), rabbit anti-HNF3 beta/FOXA2 (Millipore, #07-633), rabbit anti-PDX1 (Santa Cruz Biotechnology, #sc-25403, Santa Cruz, CA) and mouse anti-insulin (Sigma, #I2018, St. Louis, MO). Texas Red-conjugated donkey-anti-rabbit IgG (Jackson ImmunoResearch Laboratories, #711-075-152, West Grove, PA), Texas Red-conjugated donkey-anti-mouse IgG (Jackson ImmunoResearch Laboratories, #715-075-151), FITC-conjugated donkey-anti-rabbit IgG (Jackson ImmunoResearch Laboratories, #711-095-152), and FITC-conjugated donkey-anti-mouse IgG (Jackson ImmunoResearch Laboratories, #715-095-151) were used as secondary antibodies. DAPI was used for counterstaining. Stained cells were analyzed by using a confocal laser-scanning microscope (Zeiss, LSM 510 confocal scanning laser system).

### Spontaneous differentiation

For spontaneous differentiation, iPSC clones were dissociated by using collagenase IV for 30 minutes and plated on low-adhesion plates in basal HEScGRO medium without bFGF. Embryoid bodies (EBs) were cultured as a suspension for 7 to 10 days and adherent in DMEM with 20% FBS for additional 7 to 10 days. For immunofluorescence analysis, cells were fixed with 4% PFA for 20 minutes at RT. Immunostaining was performed as described earlier. Primary antibodies against FOXA2 for endoderm, beta-III tubulin (Abcam, #41489) for ectoderm, and CD31 (Santa Cruz Biotechnology, #SC1506) for mesoderm were used, whereas Texas Red-conjugated donkey anti-rabbit IgG (Jackson ImmunoResearch Laboratories, #711-075-152), and FITC-conjugated donkey anti-chicken IgG (Jackson ImmunoResearch Laboratories, #703-095-155) served as secondary antibodies.

### *In vivo *differentiation of derived iPS cells

SCID-beige mice were anesthetized, and the kidney was externalized for iPS transplantation under the kidney capsule. A small incision was made in the kidney capsule, and a blunt needle used to create a pocket under the kidney capsule. After iPSC injection into the pocket, the kidney was placed back into the abdomen, and the incision closed with a Vicryl suture. Mice were maintained for 4 weeks and killed for harvesting normal and iPS-transplanted kidneys. OTC-embedded frozen tissues were cryo-sectioned for H&E staining.

Differentiation of derived iPS cells into insulin-producing cells iPSCs were differentiated into insulin-producing cells, as reported previously with minor modifications [11]. At the first step of differentiation, human iPSC clones were treated with 25 ng/ml Wnt3a (R&D Systems) and 100 ng/ml activin A (Peprotech, Rocky Hill, NJ) in advanced RPMI (Invitrogen) with Pen/Strep for 1 day, followed by treatment with 100 ng/ml activin A in advanced RPMI supplemented with 0.2% fetal calf serum (FBS) (Invitrogen) for 2 days. At step two, cells were cultured in high-glucose DMEM (Invitrogen), supplemented with 20% (vol/vol) advanced RPMI medium containing 50 ng/ml FGF10 (R&D systems), 0.25 μmol/L KAAD-cyclopamine (CYC), and 2% FBS for 2 days. Cells were then treated with 50 ng/ml FGF10, 0.25 μmol/L CYC, and 2 μmol/L all-*trans *retinoic acid (RA) (Sigma) in high-glucose DMEM (Invitrogen) supplemented with 20% advanced RPMI, Pen/Strep, 1 × B27 supplement (Invitrogen) for 4 days at step three. Cells were then cultured in the presence of 50 ng/mL FGF10, 300 nmol/L ILV (Axxora, San Diego, CA), and 55 nmol/L GLP-1 (Sigma) in DMEM (high glucose) supplemented with 20% advanced RPMI and 1 × B27 for 4 days at step four. In step five, differentiation medium included 10 μmol/L DAPT (Sigma) and 55 nmol/L GLP-1 in DMEM (high glucose) with 20% advanced RPMI and 1 × B27, and the culture lasted for 6 days.

Finally, in step six, cells were cultured in the presence of 50 ng/ml hepatocyte growth factor (HGF) (R&D systems), 50 ng/ml insulin-like growth factor 1 (IGF-1) (R&D Systems), and 55 nmol/L GLP-1 in CMRL-1066 medium (Invitrogen) with 1 × B27 for 8 days. All differentiation experiments were performed in triplicate, and repeated at least twice.

### Microarray

Total RNA was isolated by using TRIzol (Invitrogen) and further purified by using RNeasy Plus spin columns (QIAGEN, Valencia, CA). Turbo DNA-free DNase (Ambion, Austin, TX) was used to digest all genomic DNA that could lead to false-positive gene-expression results. The RNA quantity and purity was measured with a Nanodrop spectrophotometer (Thermo Scientific, Wilmington, DE), and the RNA integrity was determined by using the Agilent 2100 Bioanalyzer (Agilent Technologies, Santa Clara, CA). Microarray analysis was performed by using the Affymetrix HG-U133 Plus2 GeneChip Array platform (Affymetrix, Santa Clara, CA). Data were preprocessed by using standard in-house MicroArray Pre-Processing workflow, and hierarchical clustering was performed by Pearson Dissimilarity. To compare the transcriptome of blood-derived iPSCs, the data set of epidermal keratinocytes (HK, SW3, SW4, and SW8), two keratinocyte-derived iPSC clones (SW3 b and SW4 N1) and human fibroblast (FB)-derived iPSC clone HCF1 [11] were used. A *t *test was performed to analyze the significance of the changes (*P *< 0.05) in the normalized gene-expression levels between HK and iPSC clones, or between blood-derived iPSC clones and HK- and FB-derived iPSC clones. Heatmap Builder software (kindly provided by Dr. Euan Ashley, Stanford School of Medicine) was used to generate the heatmap for the transcriptome data set. The registered GEO transcriptome database (GSM551202, human ES H9 cells; GSM452255, freshly isolated PBMC; GSM178554, mobilized HPCs) was used to analyze the similarities between blood-derived iPSCs and human ES cells or non-reprogrammed PBMCs and HPCs. Microarray data have been deposited in the NCBI Gene Expression Omnibus (GEO) and are accessible through GEO Series accession number GSE33536 (http://www.ncbi.nlm.nih.gov/geo/query/acc.cgi?acc=GSE33536).

## Results and discussion

### Cellular reprogramming of HPCs and PBMCs into iPSCs

**Figure 1:**
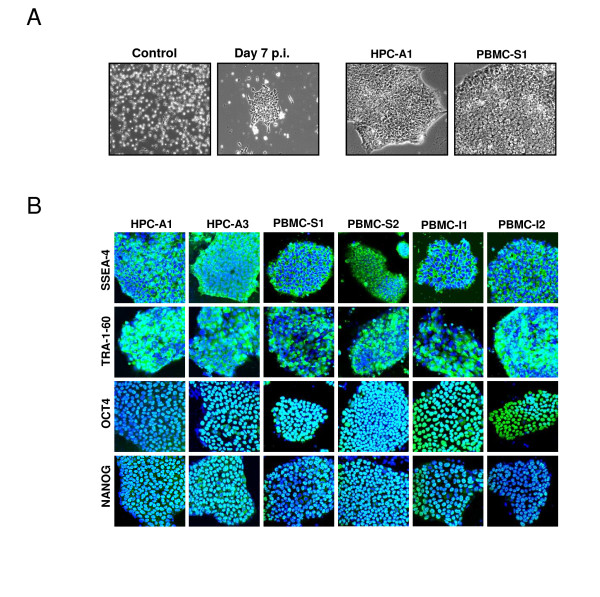
**Reprogramming of human hematopoietic progenitor and peripheral blood mononuclear cells**. **(a) **HPCs and PBMCs were cultured in a serum-free medium and transduced with lentiviral vectors expressing four stemness factors at an MOI of 5. Representative phase-contrast images of HPCs before transduction (left panel) and 7 days after infection (left panel) are shown. Representative HPC-derived (left panel) and PBMC-derived (right panel) colonies with characteristic morphologies of reprogrammed cells are shown. **(b) **HPC and PBMC-derived iPSC clones were further characterized through immunocytochemistry analysis by using a panel of antibodies against pluripotency-associated markers. All clones stained positive for the markers, including SSEA-4, TRA-1-60, OCT4, and NANOG.

**Figure 2:**
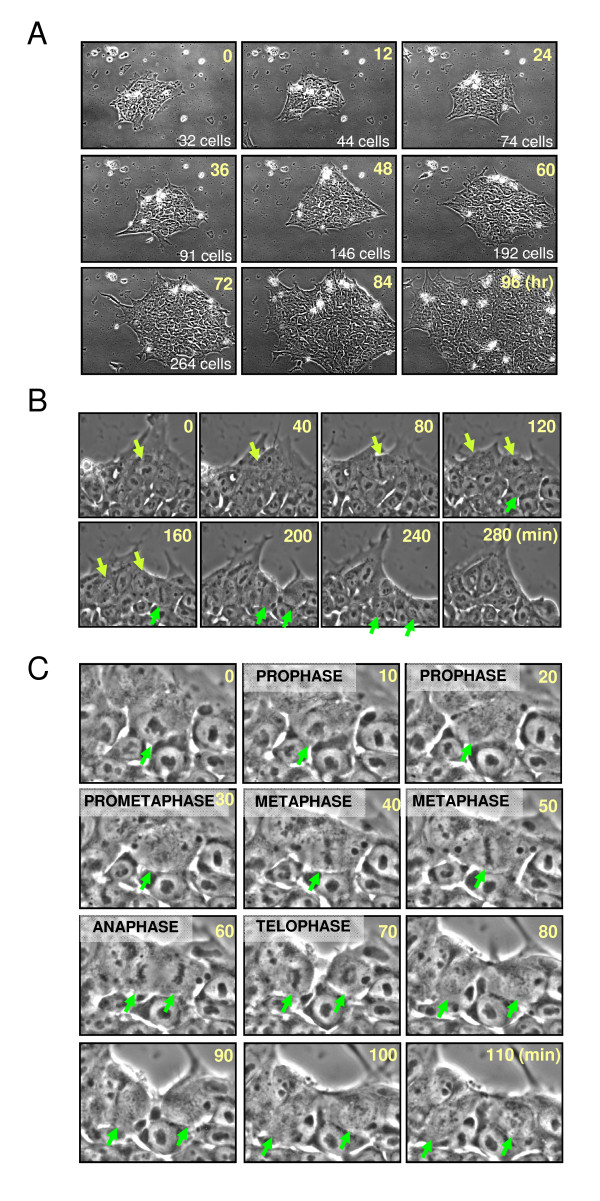
**Efficient expansion of HPC/PBMC-derived iPSC clones under feeder- and serum-free conditions**. **(a) **Long-term time-lapse images of an iPSC HPC-A1 colony were obtained by using Nikon BioStation IMQ. Time is shown in hours in the upper right corner, and cell count is shown in the bottom right corner of each panel. **(b) **Frequent mitotic events were observed during time-lapse imaging. Dividing cells and daughter cells are indicated by yellow and green arrows. Time is shown in minutes in the upper right corner of each panel. **(c) **High-magnification images of a dividing cell at different stages of mitosis (prophase, prometaphase, metaphase, anaphase, and telophase) are indicated in green arrows. Time is shown in minutes in the upper right corner of each panel.

HPCs and PBMCs were cultured overnight in a serum-free medium with CC100 cytokine cocktail (recombinant Flt-3, SCF, IL-3, and IL-6), and transduced with four stemness factor-expressing lentiviral vectors at a multiplicity of infection (MOI) of 5 each. When transduced, cells were transferred to Matrigel-coated culture plates 3 days after infection, a subset of cells attached to the plate. At 1 to 2 weeks after vector transduction, small, reprogrammed colonies, characterized by the morphology of sharp-edged, flat, and tightly packed cells, were observed (Figure 1a). No iPSC-like colony formation was observed in untransduced cells (Figure 1a). From transduced HPCs and PBMCs, we picked up 24 individual iPSC-like colonies per each sample based on their size and morphology at 2 to 3 weeks after viral transduction. Among 24 colonies, three to six colonies were expandable without spontaneous differentiation under feeder-free conditions. The reprogramming efficiency was between two to 10 expandable clones per 10^5 ^transduced cells (Figure 1b). HPC- and PBMC-derived iPS clones could be cultured for 5 months after the initial vector infection (up to passage 50) without showing signs of replicative crisis. Immunocytochemistry revealed the expression of SSEA-4, TRA-1-60, OCT4, and NANOG in the blood-derived iPSC clones (Figure 1b). Long-term time-lapse imaging demonstrated efficient iPSC expansion under feeder-free and serum-free conditions. The colonies had a 23.7-hour average cell doubling time (Figure 2a). Frequent mitotic events were observed in derived iPSC colonies (Figure 2b), and the duration of mitotic events (from prophase to telophase) was approximately 60 minutes (Figure 2b and 2c). Similar results were observed with other two iPSC clones.

### Ultrastructural studies of blood-derived iPS cells

**Figure 3:**
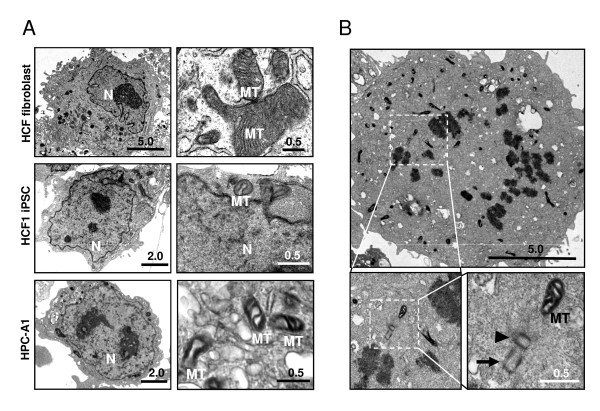
**Transmission electron microscopic images of blood-derived iPS cells**. **(a) **Representative high-resolution electron micrographs of primary human fibroblasts (HCF fibroblast), HCF-derived (HCF1 iPS), and HPC-derived (HPC-A1) iPSCs are shown. Mitochondria (MT) and nucleus (N) structures are denoted in the micrographs. **(b) **Frequent mitotic events were observed in the blood-derived iPSCs. Mother and daughter centrioles are represented by the arrowhead and arrow symbols, respectively. Scale bars are represented in micrometers.

High-resolution electron microscope analysis was performed to determine the morphologic differences between blood-derived iPSCs and verified fibroblast-derived iPSCs (HCF1) [11]. Blood-derived iPSCs showed scant cytoplasm and globular-shaped immature mitochondria with unorganized cristae, which resembled those of fibroblast-derived iPS cells (Figure 3a). In contrast, non-reprogrammed fibroblasts showed the cytoplasm densely packed with membrane-bound organelles (Figure 3a, upper left panel), including mature mitochondria with well-developed cristae (Figure 3a, upper right panel). In accordance with our cinemicrography analysis, frequent mitotic events were observed in blood-derived iPSCs cells (Figure 3b). One pair of centrioles--mother (arrowhead) and daughter (arrow) centrioles--were seen in a dividing cell at anaphase (Figure 3b, lower right panel).

### Genome-wide transcriptome analysis of blood-derived iPS clones

**Figure 4:**
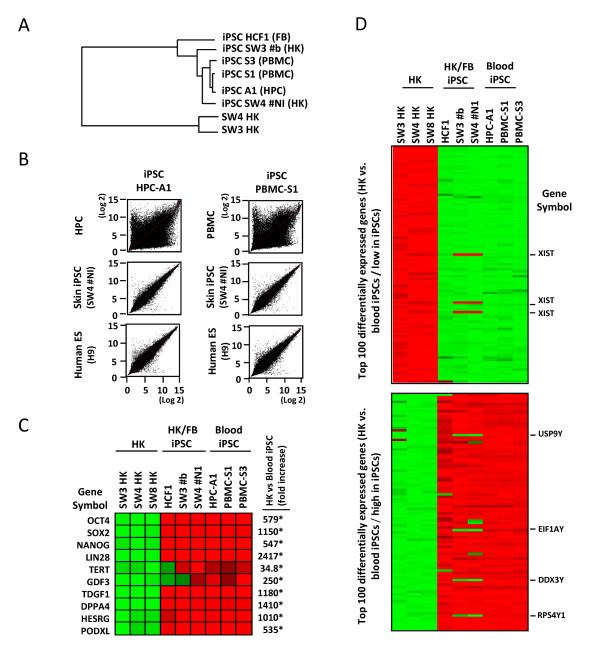
**Global gene-expression profiles of blood-derived iPSCs**. **(a) **Dendrogram describing the unsupervised hierarchal clustering of primary keratinocytes (SW3 HK and SW3 HK), and keratinocyte (HK)-, fibroblast (FB)-, and HPC- and PBMC-derived iPSCs. **(b) **Genome-wide gene-expression patterns of HPC- and PBMC-derived iPSC clones were compared with those of HPCs (GSM178554), PBMCs (GSM452255), verified epidermal keratinocyte-derived iPSCs (SW4#N1, upper panels), or embryonic stem cells (H9 cells, GSM190779). **(c) **Heatmap demonstrating the relative expression levels (high, red; low, green) of pluripotency-associated genes in primary keratinocytes (HK) and iPS cells from HK, FB, or blood cell sources. The changes in gene-expression levels in blood-derived iPSCs, relative to those in HK cells, were calculated by using the microarray data from three primary HK cells and three blood-derived iPSCs, and shown as fold-increase in iPSCs. Statistically significant changes are indicated by asterisks (*P *< 0.05). **(d) **Heatmap showing the top 100 differentially expressed genes between non-reprogrammed HK and blood-derived iPSC clones. Highly expressed in non-reprogrammed cells and blood-derived iPSCs are shown in upper and lower panels, respectively. Genes with notable differences in gene-expression patterns between HK/FB-derived and blood-derived iPSCs are indicated by the gene symbols on the right.

With a microarray representing the genome-wide transcriptome, we determined the global gene-expression patterns in HPC- and PBMC-derived iPSC clones, which were then compared with those of fibroblast (FB)- and epidermal keratinocytes (HK)-derived iPSCs. Transcriptome data from non-reprogrammed HK cells were also used as somatic cell controls. The dendrogram of unsupervised one-way hierarchic clustering analysis demonstrated that blood-derived iPSCs clustered closely with other iPSCs from different cell sources and were distinct from non-reprogrammed HK cells (Figure 4a). In accordance with this observation, the global gene-expression patterns of blood-derived iPSCs were more similar to those in human ES H9 cells and HK-derived iPSCs, rather than to non-reprogrammed HPCs or PBMCs (Figure 4b). We also noted that the transcriptome of blood-derived iPSCs was more closely related to that of HK-derived iPSCs than of ES H9 cells. This subtle difference may be due to the fact that the blood- and HK-derived iPSCs were cultured under the same feeder- and serum-free conditions in our laboratory, whereas the ES H9 transcriptome data were obtained through the GSM database. Similar to HK- and FB-derived iPSC clones, expression of pluripotency-associated genes, such as *OCT4*, *SOX2*, *NANOG*, *LIN28*, and *TERT*, was markedly upregulated in HPC- and PBMC-derived iPSC clones (Figure 4c). When the top 100 differentially expressed genes between blood-derived iPSC clones and non-reprogrammed HK cells were analyzed and used to generate heatmaps, including FB- and HK-derived iPS cells, the gene-expression patterns of blood-derived iPSCs were nearly identical to those of iPSCs derived from FB and HK cells. Among the 200 differentially expressed genes (100 highest and 100 lowest), notable differences in gene-expression profiles were found only in *XIST *(with three probes, Figure 4d, upper panel), *USP9Y*, *EIF1AY*, *DDX3Y*, and *RPS4Y1 *(Figure 4d, lower panel) in two HK-derived iPSC clones (SW3 b and SW3 NI). *XIST *is on the × chromosome, and *XIST *RNA plays a major role in silencing one of the pair of × chromosomes in female cells [36], whereas *USP9Y*, *EIF1AY*, *DDX3Y*, and *RPS4Y1 *are Y-linked genes. Because HK and HK-derived iPSC clones were from female patients, whereas HCF1, HPC-A1, PBMC-S1, and PBMC-S2 were from male patients, the observed variations in X- and Y-linked genes between blood- and non-blood-derived iPSC clones are likely due to the difference in gender of these iPSC clones.

### Pluripotency of blood-derived iPS clones verified through *in vitro *differentiation

**Figure 5:**
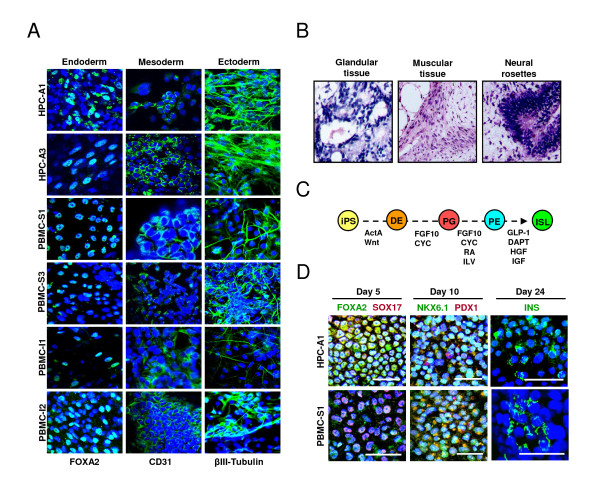
**Differentiation of blood-derived iPSCs *in vitro *and *in vivo***. **(a) **Blood-derived iPSC clones were spontaneously differentiated through embryoid body formation, and analyzed via immunocytochemistry for lineage markers for three embryonic germ layers (endoderm FOXA2, mesoderm CD31, and ectoderm β-III-tubulin). **(b) **Transplantation of iPSCs into renal capsule of SCID-beige mice resulted in teratoma formation. Tissue histology of teratomas demonstrated the cells of three germ layers including glandular, muscular, and neural rosette-like tissues. **(c) **Schematic diagram describing the stepwise-guided differentiation protocol for iPSC differentiation into islet-like cells. ActA, Activin A; CYC, cyclopamine; DE, definitive endoderm; FGF10, fibroblast growth factor 10; GLP-1, glucagon-like peptide-1; HGF1, hepatocyte growth factor-1; IGF, insulin-like growth factor-1; ILV, indolactam V; ISL, islet-like cells; PE, pancreatic endoderm; PG, primitive gut; RA, all-*trans *retinoic acid; Wnt, Wnt3a. **(d) **Through the guided differentiation protocol, HPC- or PBMC-derived iPSC clones were induced to definitive endoderm (day 5), pancreatic endoderm (day 10), and insulin-producing islet-like cells (day 24). Immunostaining demonstrated the expression of stage-specific markers in iPSC progeny at day 5 (FOXA2 and SOX17), day 10 (NKX6.1 and PDX1), and day 24 (INS). Scale bars indicate 50 μm.

HPC- and PBMC-derived iPSC clones were assayed for the ability to spontaneously differentiate *in vitro *into cells of three embryonic germ layers through embryoid body (EB) formation. All the iPSC clones assayed formed EBs. After 7 to 10 days in suspension, EBs were transferred to a Matrigel-coated plate, and spontaneously differentiated cells were expanded under adherent conditions. Immunostaining for lineage-specific markers showed that blood-derived iPSCs differentiated into cells of three germ layers, including β-III tubulin-positive ectoderm, FOXA2-positive endoderm, and CD31-positive mesoderm cells (Figure 5a).

### *In vivo *multilineage differentiation of blood-derived iPSCs

To assess the multilineage differentiation capacity of iPSCs *in vivo*, blood-derived iPSCs were transplanted under the kidney capsule of SCID-beige mice. After transplantation of 1 million cells, iPSCs formed cystic tumors within 4 weeks (Figure 3b). On gross inspection, iPSC-derived tumors demonstrated a complex cellular architecture with prominent vascularization and nonvascularized solid tissues. Histologic analysis revealed iPSC differentiation into endoderm lineages composed of glandular-like tissue, mesoderm lineages indicated by muscle-like tissue, and ectoderm lineages denoted by neural rosette-like structures (Figure 5b), which verified the multilineage differentiation capability of blood-derived iPSCs.

### Generation of insulin-producing cells from iPSCs through guided differentiation

Next we examined the pancreatic differentiation potentials of blood-derived iPSCs. We used our guided iPSC differentiation protocol with indolactam V (ILV) and GLP-1 [11], with minor modifications in medium compositions, which are described in Materials and Methods. Blood-derived iPSC clones were first stimulated with activin A and Wnt3a to form definitive endoderm cells. Immunostaining revealed the efficient induction of definitive endoderm markers SOX17 and FOXA2 in iPSC-derived cells at day 5 of differentiation (Figure 3c). Derived definitive endoderm cells were further differentiated in DMEM/advanced RPMI medium containing FGF10, CYC, and 2% FBS (vol/vol) for 2 days, and maintained in high-glucose DMEM/advanced RPMI medium supplemented with FGF10, CYC, RA, and 1 × B27 for an additional 4 days. Cells were then cultured in the presence of FGF10, ILV, GLP-1, and 1 × B27 in DMEM/advanced RPMI medium for 4 days. After this step, derived cells expressed pancreatic endoderm markers, PDX1 and NKX6.1 (Figure 5d). Further differentiation of iPSC-derived pancreatic endoderm cells was performed in DMEM/advanced RPMI medium supplemented with DAPT, GLP-1, and 1x B27 for 6 days, followed by the final maturation step in the CMRL-1066 medium containing HGF, IGF-1, GLP-1, and 1 × B27 for an additional 8 days. Insulin-positive iPSC progeny were sporadically detected (Figure 5d). High levels of intracellular C-peptide (230~320 pmol/L), a byproduct of proinsulin processing during insulin secretion, were also detected in the final differentiation-stage iPSC progeny with C-peptide ELISA (data not shown). Our results demonstrate successful differentiation of blood-derived iPSCs into insulin-expressing cells *in vitro*.

The iPSC technology opened the exciting possibility of generating patient-specific pluripotent stem cells without using an embryonic source, which could pave the way for personalized pluripotent stem cell therapy approaches. However, because of the complexity of the iPSC technology, which typically involves *ex vivo *gene delivery and pluripotent stem cell manipulation, multiple issues must be addressed before autologous iPSCs can contribute to novel individualized cell therapy. One of the major issues is to develop a GMP-compliant process for clinical-grade iPSC generation. For this end, we need simple, robust, and time-effective strategies for both iPSC derivation and differentiation. Currently, skin biopsy samples are typically used for iPSC derivation. However, it takes at least several days (often weeks) to process and expand skin cells. Here, we demonstrate the feasibility of iPSC derivation from GMP-grade mobilized HPCs and unmobilized PBMCs. Although our current HPC/PBMC iPSC derivation efficiencies (two to five clones per 10^5 ^transduced cells) were similar to those of previous reports [27,29,31,32], generation of iPSCs from GMP-grade, blood-derived cells represents an important step toward clinical applications of iPSCs and iPSC progeny cells. The use of HPCs and PBMCs enabled time-effective iPSC derivation, as the cells did not require long-term expansion before reprogramming. Moreover, apart from minor differences in global gene-expression profiles (Figure 4), blood-derived iPSCs were basically indistinguishable from iPSCs from other cell sources. Considering that many institutes/hospitals already have an FDA-approved GMP facility for autologous HPC processing, HPCs and PBMCs would be ideal somatic cell sources for clinical-grade iPSC derivation.

We and others have reprogrammed blood cells through genome-integrating viral vectors [37,38]. In our lentiviral vectors, the transgenes are driven by a retroviral promoter. Because retroviral promoter is strongly suppressed in pluripotent stem cells, we speculate that the expression of transgene-derived pluripotency factors is silenced in the blood-derived iPSCs. Nevertheless, the use of integrating vectors is associated with the risk of insertional mutagenesis and sustained/reactivated expression of reprogramming factors, including a protooncogene *c-MYC*. Indeed, stable c-Myc vector integration in iPSCs has been linked to increased tumorigenicity *in vivo *[39]. Reprogramming blood cells through non-integrating vectors or introduction of reprogramming proteins, RNAs, or miRNAs [32,40-45] would avoid/minimize these safety concerns. We are currently testing these non-integrating reprogramming strategies for patient-specific iPSC derivation.

Although islet transplantation has shown some promising results for type 1 diabetes therapy, the shortage of matched islet replacement tissues has prevented the widespread use of this therapy in the clinic. One possible solution to overcome this shortage is to regenerate transplantable insulin-secreting cells from patient-derived iPSCs [9]. We previously reported that ILV and GLP-1 facilitate differentiation of iPSCs into insulin-producing cells through a guided differentiation protocol [11]. Here we demonstrate the feasibility of generating insulin-producing cells from blood-derived iPSCs. To our knowledge, this is the first report showing the generation of insulin-producing islet-like cells from blood-derived iPSCs. In contrast to skin biopsies, which involve an invasive procedure, the use of blood cells allows minimally invasive tissue procurement for iPSC derivation. Because diabetes patients often experience poor wound healing, the minimally invasive iPSC derivation from blood cell sources would be particularly advantageous for the generation of clinical-grade iPSCs from diabetes patients.

## Conclusions

Because the use of blood cells allows minimally invasive tissue procurement under GMP conditions and rapid cellular reprogramming, mobilized HPCs and unmobilized PBMCs would be ideal somatic cell sources for clinical-grade iPSC derivation, especially from diabetes patients complicated by a slow-healing wound.

## Abbreviations

CYC: cyclopamine; EB: embryoid body; ES: embryonic stem; FB: fibroblast; FBS: fetal bovine serum; FDA: Food and Drug Administration; HCF: human cardiac fibroblast; HGF: hepatocyte growth factor; HK: human keratinocyte; HPCs: hematopoietic progenitor cells; IGF-1: insulin-like growth factor 1; ILV: indolactam V; iPSC: induced pluripotent stem cell; PBMCs: peripheral blood mononuclear cells; RA: retinoic acid.

## Competing interests

TT, YK, and YI are currently preparing a patent application with the Mayo Clinic. The other authors have no competing interests.

## Authors' contributions

SO, YK, and YI designed the study, carried out the time-lapse imaging, electron microscopy, and molecular genetic studies, and drafted the manuscript. ABD and DRM provided and verified the GMP-grade cells. MCD carried out the teratoma-formation assay. KAH reprogrammed blood cells and helped to draft the manuscript. TT established the guided differentiation protocol for insulin-producing cells. TS generated reprogramming vectors. All authors critically read, revised, and approved the final manuscript.
